# Study on Fatigue Characteristics of Bionic Functional Surface of Hardened Steel

**DOI:** 10.3390/ma13184130

**Published:** 2020-09-17

**Authors:** Youzheng Cui, Minli Zheng, Wei Zhang, Ben Wang, Yonglei Sun, Weiran Wang

**Affiliations:** 1 Key Laboratory of Advanced Manufacturing and Intelligent Technology, Ministry of Education, Harbin University of Science and Technology, Harbin 150080, China; cuiyouzhenghust@163.com (Y.C.); minli@hrbust.edu.cn (M.Z.); wangbenhust@163.com (B.W.); sunyonglei18201@163.com (Y.S.); 15245201103@163.com (W.W.); 2 School of Mechanical and Electronic Engineering, Qiqihar University, Qiqihar 161006, China

**Keywords:** hardened steel die, high-speed milling, biomimetic function surface, fatigue life analysis, crack propagation

## Abstract

In this study, we aimed to process the biomimetic function surface by designing a prototype for modeling the pits on a dung beetle body and the abdomen of a desert viper, and by using high speed milling and controlling the ratio of row spacing to feed rate. Firstly, we conducted three-dimensional parametric modeling and static analysis of the bionic functional surface using 3D modeling software UGNX (12.0, SIEMENS AG, Munich, Germany) and finite element analysis software ABAQUS (2018, Dassault, Providence, RI, USA). Then, the analysis results were imported into the fatigue life analysis software nCode (2018, HBM United Kingdom Ltd., South Yorkshire, UK) to simulate the fatigue characteristics of different bionic pit morphology models. Per the simulated tensile fatigue testing machine, the result shows that the minimum fatigue life value of the quadrilateral pit surface of the simulated dung beetle is one and four times higher than the hexagonal pit morphology and the irregular pit morphology, respectively, whereas the maximum fatigue damage is lower by one and five orders of magnitude, respectively. The quadrilateral pit surface on the biomimetic dung beetle body has better fatigue resistance, which can considerably improve the fatigue damage distribution state and the fatigue life of hardened steel die surfaces. The influential regulation of milling parameters on fatigue performance was studied and the results show that the fatigue resistance of the model is optimal when milling parameters are: row spacing of 0.4 mm, loading space of 0.2 mm, and milling depth of 0.3 mm. The quadrilateral dimensions formed by milling are highly similar to those of a dung beetle body proving that a certain reduction in milling process depth can increase the structural fatigue resistance. From the perspective of fatigue crack growth analysis, the quadrilateral dimples on the surface of the dung beetle improve fatigue crack growth inhibition and fatigue resistance.

## 1. Introduction

Hardened steel is a material commonly used for manufacturing panel dies on automobiles. The processing surface of hardened steel die after high-speed milling basically reflects its surface state in its final form, and its processed surface state directly influences the die life span [1]. Automobile panel dies are repeatedly subjected to compressive stress and friction during the process of press forming, which easily produces defaults like pits, abrasion, and scratches on the die surface. Fatigue cracks on the model surface can easily generate in the long term, which may cause die failure or scrap, which result in economic loss. Therefore, die performance must be improved to extend its service life span.

Fatigue resistance, wear resistance, drag reduction, anti-adhesion, lubrication, and other characteristics on some organisms or objects in nature [2,3,4,5,6] can provide inspiration for improving die fatigue resistance and wear resistance. Many studies have been conducted to improve fatigue resistance by introducing bionics technology into die application. Ma et al. [7,8], inspired by bionics, introduced a crack repair method using discontinuous laser melting to fix thermal fatigue cracks on a retarding disk, which extended the life span of the retarding disk and increased repair efficiency. Lu et al. [9] studied the fatigue life of thermal cracking on H13 tool steel by generating thermal cracks through repeated heating and cooling based on traditional invasive testing. A finite element model was established to determine the thermal load and the result agreed with experiment measurements. A prediction principle based on temperature was proposed to depict thermal fatigue crack life, which could be obtained from a revised general gradient equation. Liu et al. [10] strengthened the die casting mold surface by biomimetic strengthening the die via coupling laser surface melting and laser surface filling technologies. The thermal fatigue crack extension forms under different treatment methods were analyzed and the result showed that a biomimetic unit’s hardness when treated by laser is higher than when treated by laser surface melting; the sample treated by laser surface filling had higher thermal fatigue resistance. Zhang et al. [11], by studying soil animals, introduced a bionic theory of non-smooth surfaces. After applying the theory to dies in operation, the thermal fatigue resistance was improved. Jia et al. [12] strengthened H13 steel using the laser surface melting method and discovered that laser biomimetic units can increase die life span. Cong et al. [13,14] researched the thermal fatigue resistance changes of H13 steel under two treatments: laser alloying and laser melting. Meng et al. [15] produced a non-smooth surface of annealed H13 steel using laser cladding and compared the fatigue performance of samples. Tong [16] and Daim [17] combined non-smooth surface forms on shells and applied biomimetic non-smooth surface forms to the surface of grey cast iron using laser processing, and the result demonstrated the efficiency of improving material fatigue resistance using this method. Shan et al. [18] used the non-smooth shape, structure, and function of an animal body surface as a biological prototype and used laser processing to reduce the bionic non-smooth unit on the mold surface, which significantly reduced the adhesion of the mold surface. Yin et al. [19] the fatigue crack repair of Cr12MoV die was studied using laser wire-filled cladding technology. Meng et al. [20] the relationship between the mechanical properties and thermal fatigue behavior of the materials treated by selective laser surface melting was discussed by combining bionic theory and laser technology. Yang et al. [21] after laser bionic coupling technology (LBCT) was used, the thermal fatigue of the thermal processing mold significantly improved, and the effect of preventing the initiation and propagation of cracks is significant. In Zhi et al. [22] a coupling bionic model combination was prepared on the mold surface by laser re-melting bionic technology and the fatigue crack number and wear loss weight of the coupled bionic model were compared at different temperatures. The mechanism of the bionic coupling model was analyzed, and it was determined that the bionic surface with a dense row structure had strong fatigue wear resistance. Zhao et al. [23] by using appropriate laser process parameters, a grid-like bionic non-smooth coupling unit can be prepared on the surface of the H13 steel hot forging die. The cross section of the unit body is a convex hull-shaped unit structure; it is hot forged with H13 steel without a surface treatment. Compared with the mold, the bionic non-smooth coupling treatment significantly improves the surface hardness, wear resistance and thermal fatigue performance of the mold. Han et al. [24,25] the design and manufacture method of the bionic functional surface of a scorpion is proposed to improve the corrosion resistance of parts in environments with dust and sand. Tillmann et al. [26] the honeycomb-like functional surface structure of an imitation scarab beetle head was processed by laser micro-processing technology. Compared with a polished surface, the bionic structure has a lower friction coefficient, which increases the wear resistance required by the structure.

**Figure 1:**
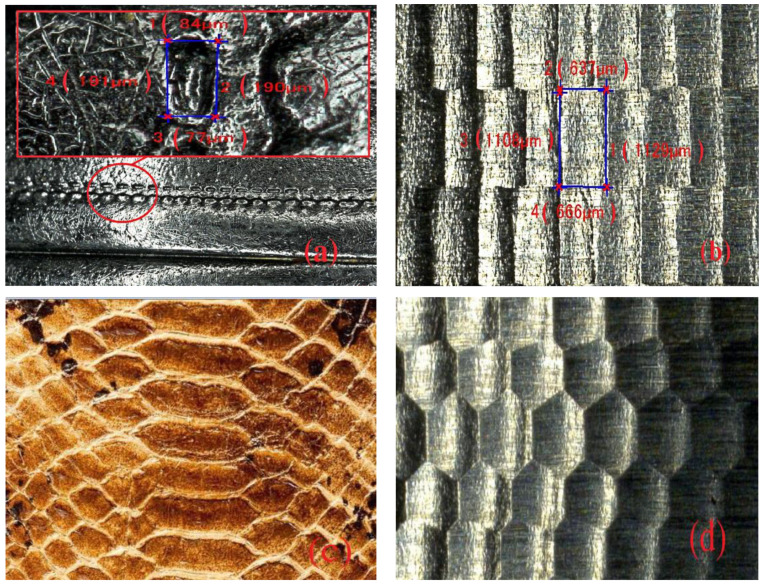
Different biomimetic designs and non-smooth pit surface forms. (**a**) Biomimetic design of quadrilateral pit on dung beetle body; (**b**) biomimetic form of quadrilateral pit after high-speed milling; (**c**) biomimetic design on abdomen of desert viper; (**d**) biomimetic form of hexagonal pit after high-speed milling.

Based on the literature review, most researchers have focused on the thermal fatigue resistance of dies and their biomimetic strengthening treatment on the mold surface based on different bionic forms using laser melting technology to improve the thermal fatigue resistance and die lifespan. At the same time, research on bionic functional surface has primarily aimed to improve the structure’s resistance to wear and corrosion. However, research examining the biomimetic fatigue of cold dies on large automobile panels is lacking. In the manufacturing of automobile panel dies, especially hardened steel dies with complicated shapes and large dimensions, the laser surface strengthening methods are unsuitable because large, precise, and complicated automobile panel dies are unquenched before the overall high-speed milling. If the surface is treated one more time after milling, the formed surface hardening layer will be thin and its adhesion with basic materials will be weak, which will wear easily during service, thereby increasing manufacturing expense and the manufacturing period, which influence the development and manufacturing of automobile panel dies. Inspired by bionic organisms with biomimetic non-smooth surfaces on typical organism bodies like dung beetles and desert vipers, in this study, we used the hardened steel pitting style biomimetic function surface produced by high-speed milling technology (Figure 1) to construct an effective method for improving die performance and increasing life span, which has value and practical significance for enterprises to reduce production costs, improve productivity, and widen the application range of drag reduction, wear resistance, and fatigue resistance on non-smooth surfaces.

## 2. Modeling and Simulation Procedures

### 2.1. Fatigue Cumulative Damage Theory

Fatigue analysis is mainly based on fatigue cumulative damage theory and is an important method for predicting the safe fatigue life under different alternating stress amplitudes. The structure starts to experience cumulative damage at defaulted spots when bearing stress load above the fatigue limit, which results in breaking above over the material bearing limit [27]. We adopted Palmgren-Miner [28] linear cumulative damage theory. The structural fatigue damage is mainly determined by loading frequency and loading amplitude value. The damages produced under different loadings are independent and can be accumulated. The total damage is the sum of each loading amplitude value:

(1) $$D=\sum_{i=0}^{k}\frac{n_{i}}{N_{i}}$$

where: *D* is the structural total fatigue damage, *k* is the stress level of variable amplitude load, *N_i_* corresponds to the fatigue life under *i-*th stage loading, and *n_i_* is the cycling times of the loading at number stage *i*.

(2) $$D=\sum_{i=1}^{N_{f}}\frac{n_{i}}{N_{i}}=\sum_{t\leq T}^{}\frac{1}{N_{sk}}=D_{f}$$

where: *N_sk_* indicates that time *t* corresponds to the number of cycles corresponding to stress amplitude *S_k_*, and *T* is the time sum after the completion of all cycles.

### 2.2. Velocity Theory Model of Fatigue Crack Extension

We adopted the Paris equation [29], commonly used in engineering to precisely depict the velocity of the crack extension as well as to evaluate the fatigue crack extension life in the medium speed rate stage, the calculation equation is as follows:

(3) $$\frac{da}{dN}=C{\left(\Delta K\right)}^{m}$$

where: *a* refers to crack length, *N* refers to the number of stress cycles; *da*/*dN* is the crack extension velocity; *C* and *m* are both mean material constant; and Δ*K* means magnitude of stress intensity factor [30].

(4) $$\Delta K=K_{max}-K_{min}=f\Delta \sigma \sqrt{\pi a}$$

where: *f* refers to the structural geometry and crack dimension coefficient; *K_max_* and *K_min_* mean maximum and minimum stress intensity factor, respectively; Δ*σ* is the stress amplitude value at cracking point.

### 2.3. Morphology Modeling and Simulation

#### 2.3.1. 3D Modeling and Static Analysis of Different Bionic Shapes

The ball-end milling cutter moves along a prescribed path while rotating, to remove of material with the rotation and translation of the tool, and finally form a new surface. The modeling, was represented by the Boolean operation of the tool to find the difference on the workpiece. To ensure the similarity of the pits on the model surface to the actual surface shape after high-speed milling, three types of bionic pits, quadrilateral, hexagonal, and irregular chaotic pits were processed by high-speed milling with 3D modeling software UGNX (12.0, SIEMENS AG, Munich, Germany). The pit morphology was modeled parametrically. Considering the computing power and the efficiency of the finite element solution, the size of the workpiece was adjusted and the number of pits was reduced proportionally. The size of the model was 4 × 2 × 1 mm.

**Figure 2:**
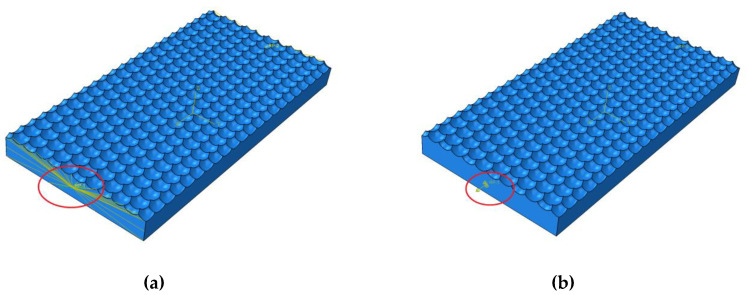
Constraints and loads imposed by the model: (**a**) constraints imposed, (**b**) application of load.

**Figure 3:**
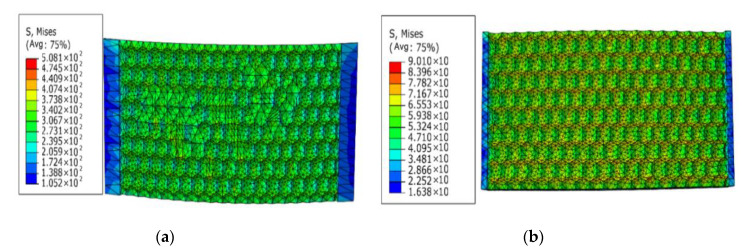

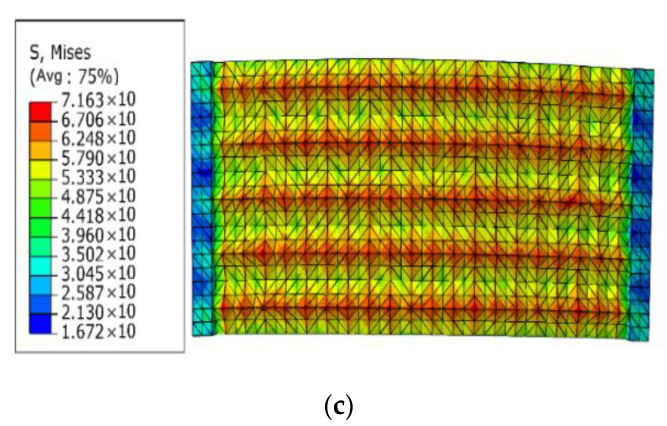
Static stress distribution nephogram of three different bionic shapes: (**a**) irregular, (**b**) hexagonal surface form, and (**c**) quadrilateral surfaces.

We selected Cr12MoV hardened steel as the die material; its chemical composition is shown in Table 1 and its physical characteristics in Table 2. We imported the drawn 3D model into the finite element analysis software ABAQUS (2018, Dassault, Providence, RI, USA) including setting material parameters, meshing, and applying constraints. The shape and number of grid divisions directly affect the accuracy of model analysis. Hexahedral mesh is preferred for drawing, but due to the surface curvature of hexagons and the irregular topography being too large, we could not successfully seed the hexahedral mesh, so tetrahedral mesh was selected for these two topography, and hexahedral mesh is used for quadrilateral model. In this paper, the fully integrated C3D8 (3D solid element with eight nodes) element type is adopted. At the same time, in order to ensure the analysis accuracy, the number of all model grids should be set at more than 15,000. In terms of setting constraints, first create the central node on the two sides of the model and use RP (Reference Point) to create reference points. The constraint is defined as MRP (The way to constrain) beam unit, which completes the creation of model loading point. The model is set as MPC (Multi-point constraints) constraint, as shown in Figure 2a. In terms of load setting, based on the RP reference point created previously, the displacement boundary conditions are selected. The displacement of the model in the Y and Z directions is set to zero, and a pair of concentrated forces with the size of 20 N are applied on the reference point RP, and the direction points to the outward normal direction along the X direction. The specific load setting is shown in Figure 2b. After analysis, the static stress distribution nephogram of three morphologies is obtained, as shown in Figure 3.

From the static stress simulation analysis results of the three different surface models, we found that the stress distribution of the irregular surface was the worst. The maximum surface stress value of the model under static tension reaches was 508 MPa, and the maximum stress point distribution was irregular and more concentrated, basically appearing at the junction where the shape of the surface pit changed. Compared with the irregular morphology, the maximum stress of the hexagonal morphology was 90 MPa, and the distribution was more uniform, appearing at the bottom of the pit and the cusp of the surface where the pit was connected. The static stress distribution of the quadrilateral morphology was more uniform and regular than that of the irregular and hexagonal morphologies, being evenly distributed at the cusp of the pit surface. We observed no obvious stress concentration in a few points, and the maximum static stress was only about 70 MPa, which is less than the maximum stress value of the hexagonal model and far lower than the maximum stress value of the irregular surface topography.

#### 2.3.2. Fatigue Analysis of Different Bionic Morphology Models

**Figure 4:**
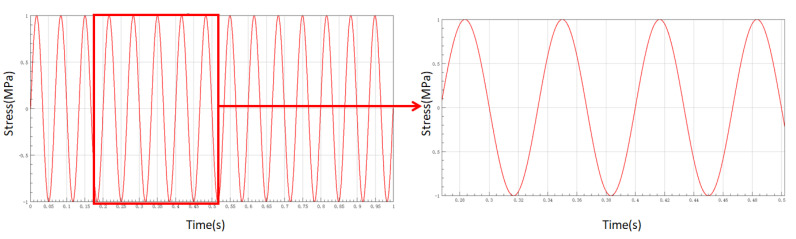
Fatigue life analysis load spectrum.

According to the actual working conditions of the simulated fatigue tensile experiment, we aimed to determine the load loading mode and load frequency. Considering the calculation capacity of the computer and the efficiency of the finite element solution in the previous section, the load was scaled proportionally according to the actual situation and model requirements. The tensile force was set to 20 N, the frequency to 15 Hz, and the waveform was set to sinusoidal. The load spectrum is shown in Figure 4.

**Figure 5:**
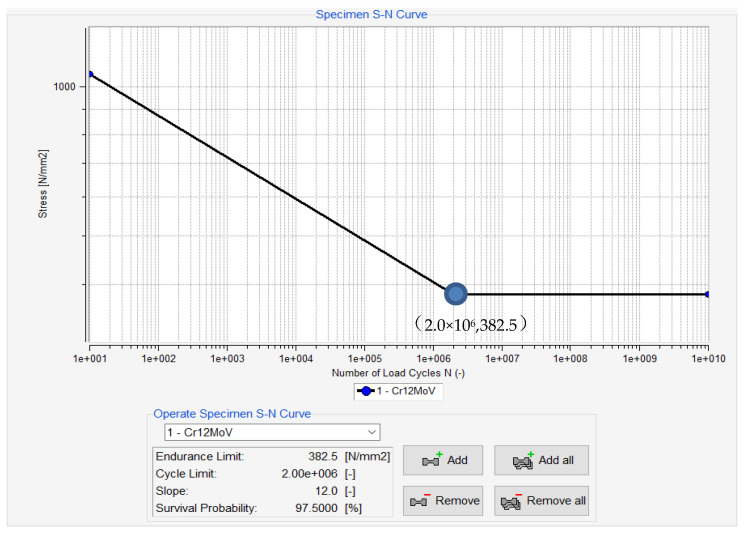
S-N curve of Cr12MoV.

Different structural forms of components have different degrees of influence on fatigue. Measuring the fatigue characteristics of different components according to the actual situation, is too complicated and cumbersome, so the material is used as the basis for analyzing the fatigue characteristics of the material. During this process, adding a correction factor to different components can produce results that meet the actual situation. The S-N curves of common materials are provided in many fatigue analysis softwares. The S-N curve of Cr12MoV used in this paper was obtained using fatigue analysis software nCode (2018, HBM United Kingdom Ltd., South Yorkshire, UK), as shown in Figure 5.

**Figure 6:**
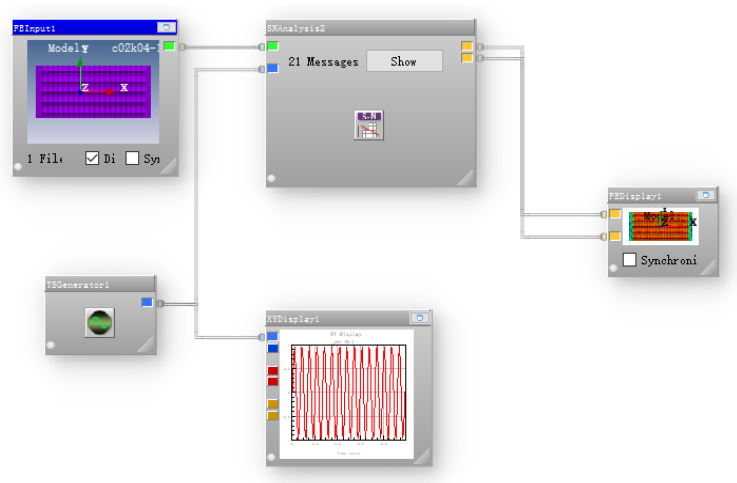
Flow chart of nCode fatigue analysis.

The odb format file with the static analysis results was imported into the nCode fatigue analysis software, and the analysis process (as shown in Figure 6) is established. The load frequency was 15 Hz, sine wave was selected, and the material fatigue characteristic S-N curve was transferred to complete the numerical simulation of the fatigue life of the three groups of different bionic functional surface models. The surface fatigue resistance was selected as the basis for further research on the highest surface fatigue resistance.

## 3. Fatigue Value Simulation Results and Discussions

### 3.1. Comparative Analysis of Fatigue Features of Three Biomimetic Forms

**Figure 7:**
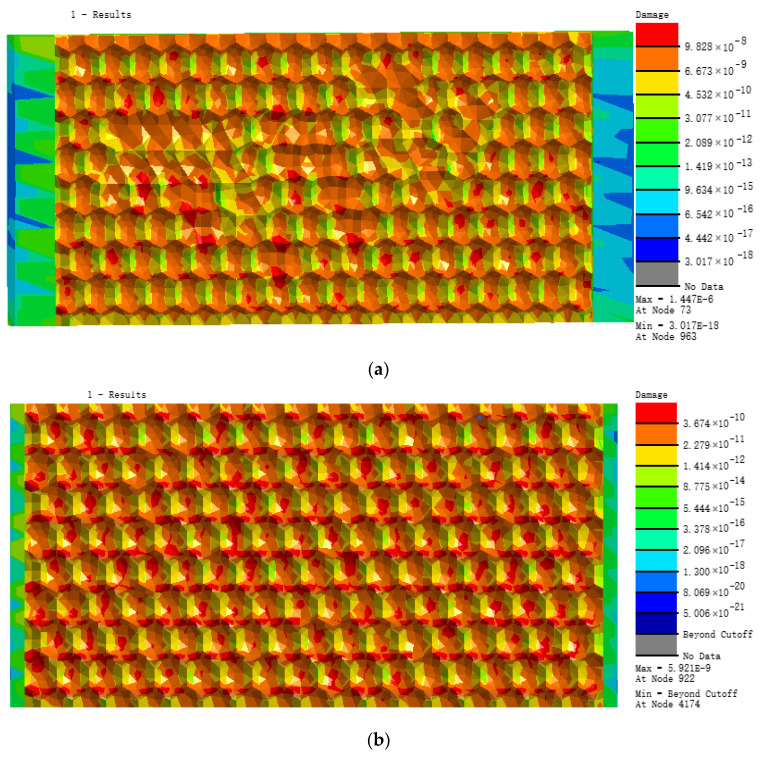

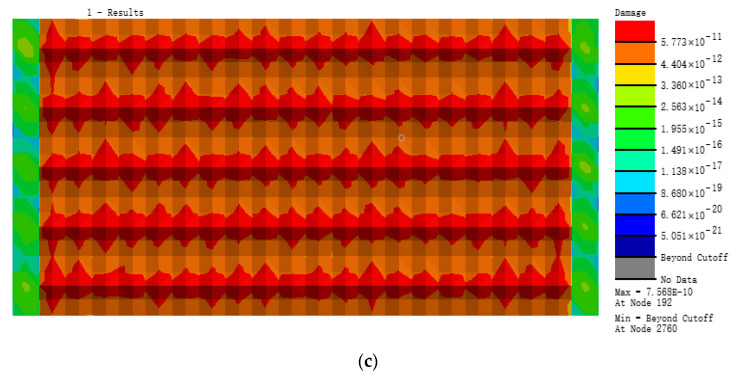
Nephograms of fatigue damage for three models: (**a**) irregular surface, (**b**) hexagonal surface, and (**c**) quadrilateral surface models.

According to the load loading mode and frequency determined by the simulated fatigue test, the research object in this paper selects the tensile force as 20 N, the frequency as 15 Hz, and the sine wave as the waveform. Under this load level, three different bionic profile models are obtained. Fatigue damage cloud diagram (shown in Figure 7). The fatigue damage on three different biomimetic form models is shown in Figure 6. The fatigue damage on the irregular biomimetic form model concentrated at the joints of various pits with a maximum damage at 9.808 × 10^−6^, but little damage to other parts of the surface was found, which indicated the concentration under an alternating load. The damage for the hexagonal model was evenly distributed and the maximum damage, at 5.921 × 10^−9^, mainly appeared at the surface cusp where two pits meet. The damage for the quadrilateral model was the most evenly distributed among the three models and the damage inside the pit was minimal, being mainly found at the surface cusp with maximum fatigue damage of 7.568 × 10^−^^10^.

**Figure 8:**
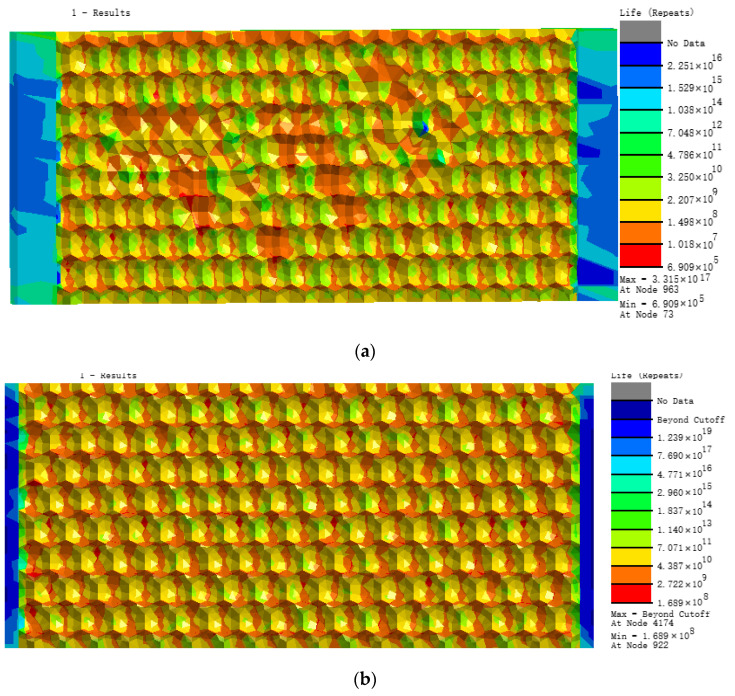

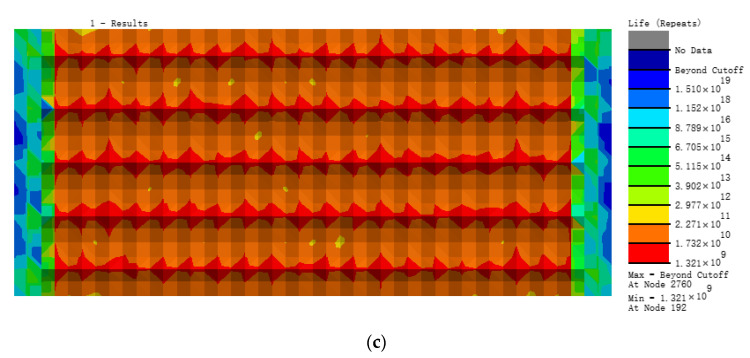
Nephograms of fatigue life of three models: (**a**) irregular surface, (**b**) hexagonal surface, and (**c**) quadrilateral surface models.

Figure 8 shows the distribution in nephograms of fatigue damage for the three biomimetic form models. For the irregular surface form, the model fatigue life at joints of different pits under alternating stress was the lowest, only 6.909 × 10^5^ times, due to the serious stress concentration. The minimum fatigue life of the hexagonal form model was 1.698 × 10^8^ times and that of the quadrilateral surface form model was 1.321 × 10^9^ times, which is superior to the former two biomimetic forms.

**Figure 9:**
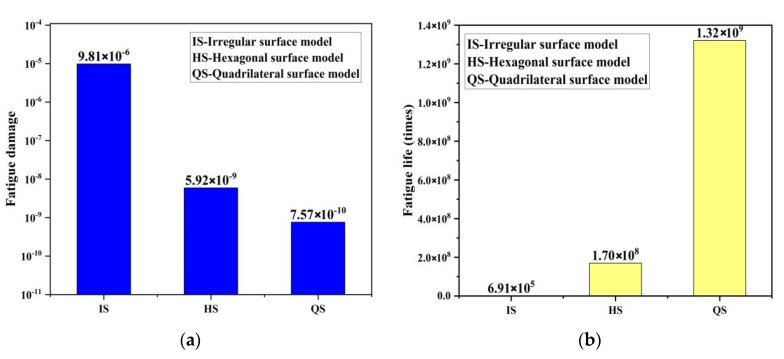
Comparison of fatigue features of three different form models: (**a**) fatigue damage, and (**b**) fatigue life.

The nephograms of the fatigue damage distribution in Figure 7 and the comparison of the fatigue features in Figure 9, show that the irregular pit surface is a micro-form surface with the worst fatigue features, which is commonly observed with high-speed milling; therefore, subsequent research using the irregular surface form model was abandoned. The fatigue features of the hexagonal surface model were three orders of magnitude higher than those of the irregular surface model and there was also an obvious concentration of fatigue damage at the bottom of the pits and joints of surfaces. The fatigue life of the quadrilateral surface model was the longest compared with the other two models and its fatigue damage distribution was more even than that of the hexagonal surface model. So, based on the results and comparisons of the three different surface micro biomimetic forms, we conclude that the quadrilateral pit surface biomimetic form has the optimal fatigue resistance, which was the dung beetle surface pit form.

### 3.2. Fatigue Analysis of Quadrilateral Biomimetic Form with Different Milling Parameters

**Figure 10:**
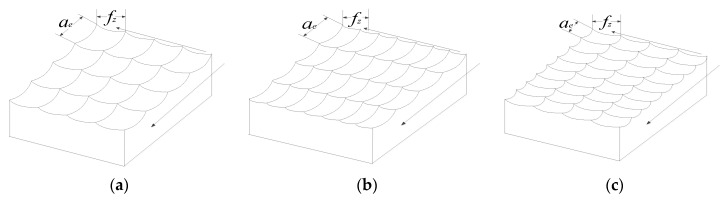
Arrangement of micro units.

The micro-form arrangement on the processing surface is shown as quadrilateral pits formed by four curves using ball-nose cutters. The feature parameters of the micro-units, like length and depth, are affected by the feed rate per tooth, row spacing, radius of the ball-nose cutter, and milling depth during milling. The number of micro-unit pits increased gradually when the ball-nose cutter moved along feeding direction and the processing surface form was completed after the ball-nose cutter high-speed milling, as shown in Figure 10. By controlling the ratio of row spacing to feed rate, a biomimetic function surface with higher fatigue resistance was produced through alternating process parameters. The a type square pit form was formed when the ratio of row spacing to feed rate was approximately one, which means the row spacing was equal to the feed rate; the b and c types are rectangular pits formed by different ratios of row spacing to feed rate. As a result, the modeling of quadrilateral pit form by changing the ratios of row spacing and feed rate, and the fatigue feature analysis can be used to predict the optimized processing parameters of fatigue resistance, which builds the research foundation for guiding the preparation of biomimetic function surfaces with increased fatigue resistance.

#### 3.2.1. Fatigue Features of Models with Row Space Changes

The feed row spacing *f_z_* and milling depth *a_p_* of the first set of models were fixed and row spacing *a_e_* was varied. The specific parameters are shown in Table 3.

**Figure 11:**
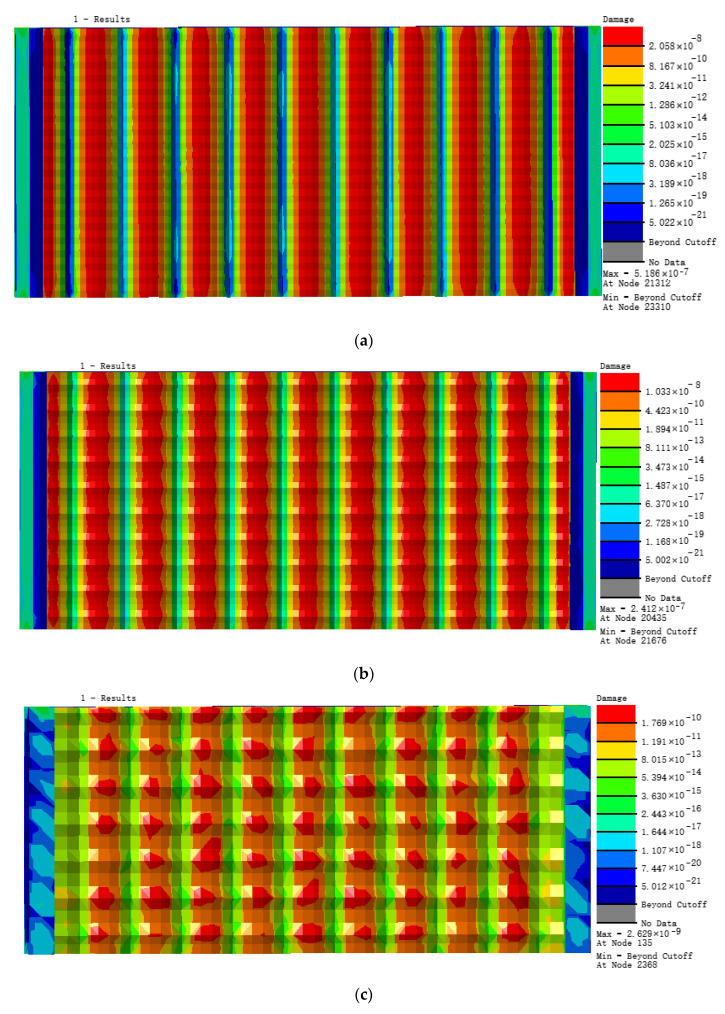

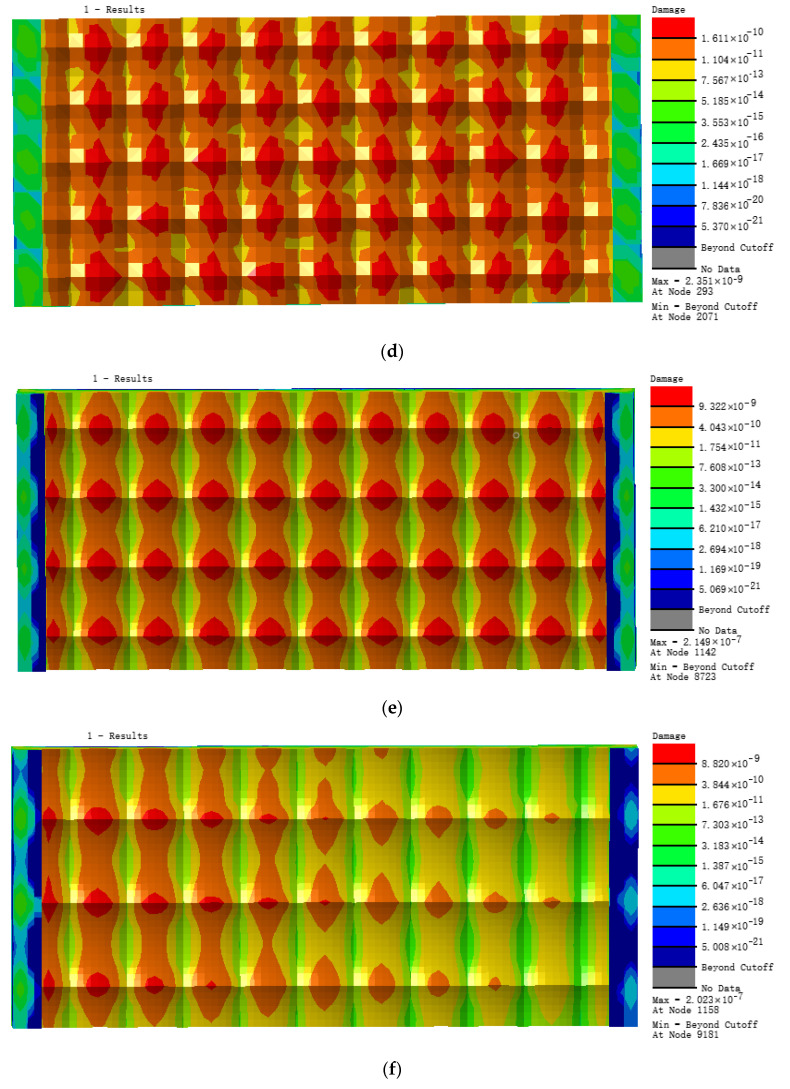
Nephograms of fatigue damage. (**a**) A1 model; (**b**) A2 model; (**c**) A3 model; (**d**) A4 model; (**f**) A5 model; (**e**) A6 model.

From the analysis of the distribution nephograms of the fatigue damage of six models in Figure 11, we found that the distributions of fatigue damage of all six models gradually transferred to the peak point of residual height along the uphill edge arc from the concentrated points in the pit bottom with increasing of row spacing. The damage was scattered evenly at the surface cusps in model A4, followed by the gradual transfer to the middle and bottom parts of pits along the downhill edge arc after crossing the surface cusps. The fatigue damage of former two models concentrated in a linear distribution at the pit bottom, which showed the concentration of stress at the pit bottom. The fatigue damage of model A4 was most distributed, with similar damage over the whole surface, which indicated that the whole surface was stressed evenly by loading and stress did not concentrate in the pit bottom. Model A6 was different from the other models, showing damage concentrated on one side of the model and that continued growing from right to left and from low to high.

**Figure 12:**
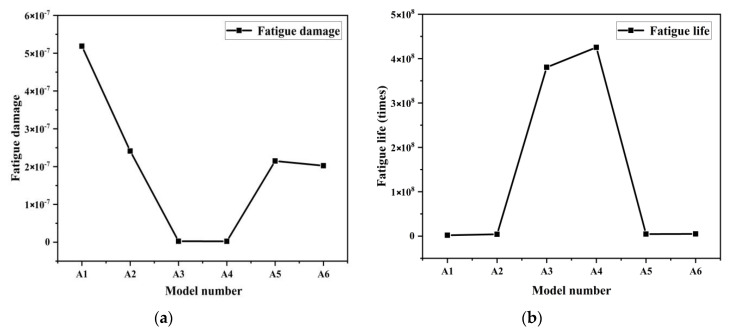
Variation curve of (**a**) fatigue damage and (**b**) fatigue life of the six models.

Figure 12b shows that the fatigue life of model A1 (number of cycles that bear loads) was 1.928 × 10^6^ times, which was the lowest among the six models. The specific cloud diagram of fatigue life distribution can be seen in Figure S1. The variation in the curve of fatigue life reflects the trend of first increasing then decreasing. It reached the maximum of 4.253 × 10^8^ in model A4. The variation in the trend of fatigue damage in Figure 12a is reciprocally related to the variation in fatigue life, and the minimum and maximum values of fatigue damage correspond to the maximum and minimum fatigue life values, respectively. For the quadrilateral biomimetic form, when the long side of the quadrilateral was parallel to the load line, the fatigue life of the model increased with the decreasing ratio of row spacing to feed rate (length to width). Model A4, with a ratio of length to width of 1:1, had the longest fatigue life and least fatigue damage at 2.351 × 10^−9^, then the fatigue damage of the model increased gradually with increasing of length-width ratio. Under a fixed feed space *f_z_* but variable row spacing *a_e_*, the smaller the ratio of length to width, the higher the fatigue resistance. Therefore, the processing parameters of model A4 with ratio of length to width of 1:1 was the best parameter combination scheme in this set of models.

#### 3.2.2. Fatigue Features of Models with Changing Feed Space

The feed row spacing *a_e_* and milling depth *a_p_* of the second set of models were fixed and, feed space *f_z_* was varied. The specific parameters are shown in Table 4.

**Figure 13:**
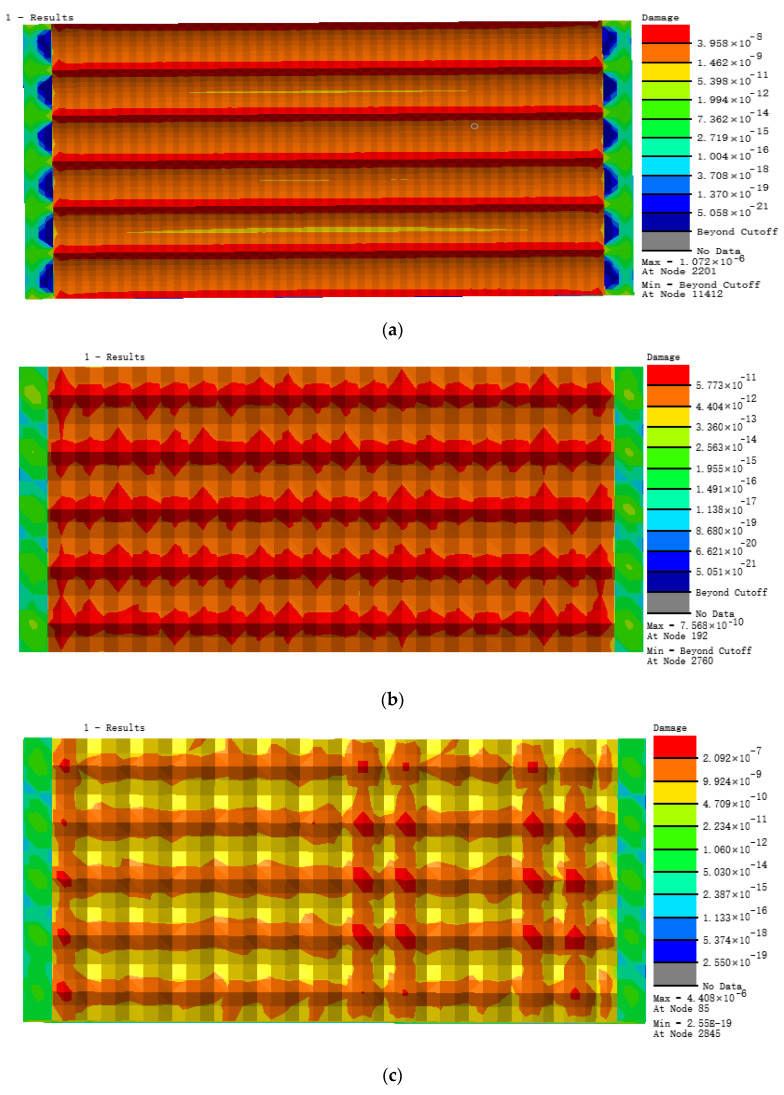

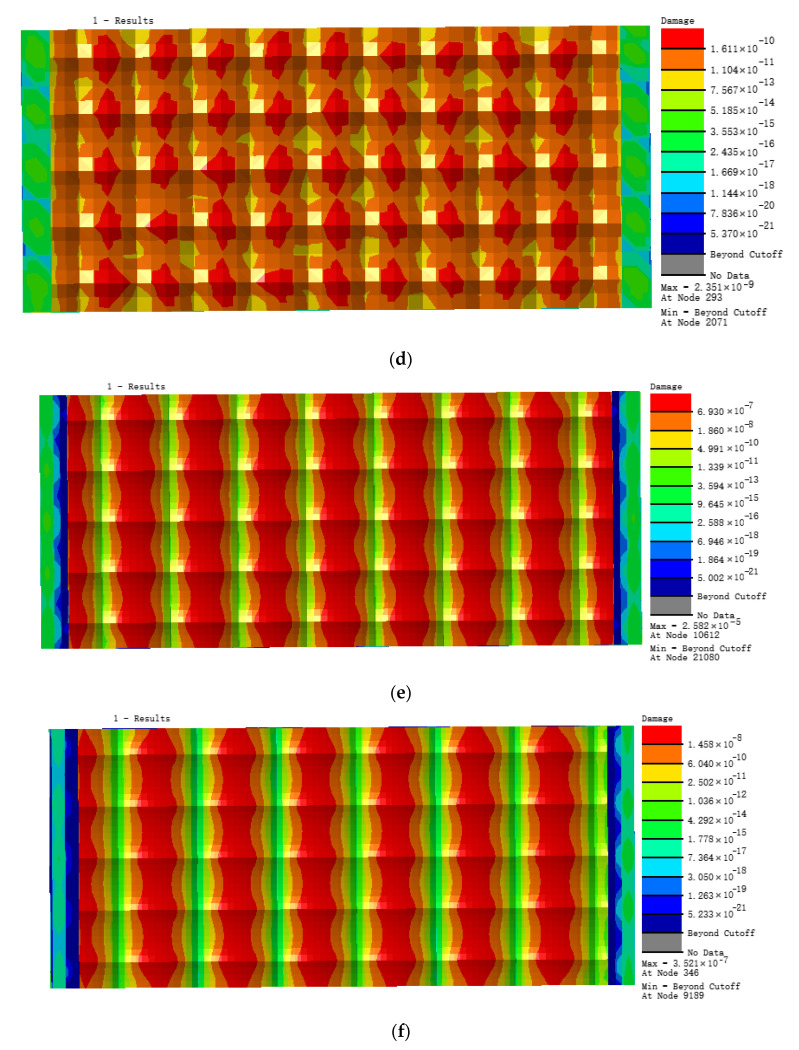
Nephograms of fatigue damage. (**a**) F1 model; (**b**) F2 model; (**c**) F3 model; (**d**) F4 model; (**f**) F5 model; (**e**) F6 model.

From the analysis of nephograms of the fatigue damage for the six models in Figure 13, the fatigue damage distribution linearly concentrated in the ridge peak area on the pit surface with increasing feed space, and then transfered to the pit bottom along the ridge on both sides of the peak. Fatigue damage at the surface peak cusps was evenly scattered on model F2, which then transferred to model F6, where large area of damage concentrated with a linear distribution at the pit bottom, which negatively influenced fatigue features, showing obvious stress concentration at the pit bottom under alternating load cycling. Fatigue damage on model F5 had a maximum value of 2.582 × 10^−5^, where the even fatigue damage distribution state positively influenced the fatigue features, indicating the lack of obvious stress concentration for the model under exterior load. The fatigue damage on model F2 had the lowest value, at 7.568 × 10^−10^, of this set of models.

**Figure 14:**
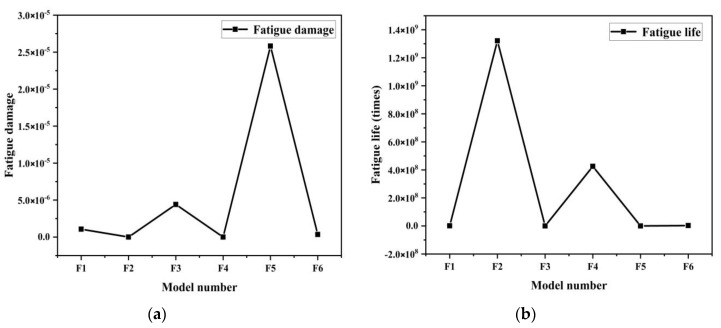
Variation curves of (**a**) fatigue damage and (**b**) fatigue life of six models.

Figure 14b that shows that the fatigue life of model F2 was the longest at 1.321 × 10^9^ times load cycles. The fatigue life of model F5 was the shortest, with only 3.872 × 10^4^ times. The specific cloud diagram of fatigue life distribution can be seen in Figure S2. By analyzing the variation in the curve of fatigue damage of the six models, we found that of model F2 was the lowest among the six at 7.568 × 10^−10^, which was found to be the optimal parameter combination for fatigue resistance.

Through comparison and analysis of the fatigue damage and fatigue life of the two best models, model A4, with a length-width ratio of 1:1 and model F2 of 2:1, we discovered that the minimum fatigue life of model F2 was 210.6% higher than that of model A4, whereas its maximum fatigue damage value was 67.8% lower than that of model A4. In summary, model F2, with a length-width ratio of 2:1, has the optimal model size for fatigue resistance, which agrees with the length-width ratio of the biomimetic form of dung beetle body, as shown in Figure 1.

#### 3.2.3. Fatigue Features of Models with Changing Milling Depth

To determine the influence of milling depth on the optimal fatigue resistance of the selected surface forms, four sets of models with different milling depths were introduced, their specific parameters are shown in Table 5.

**Figure 15:**
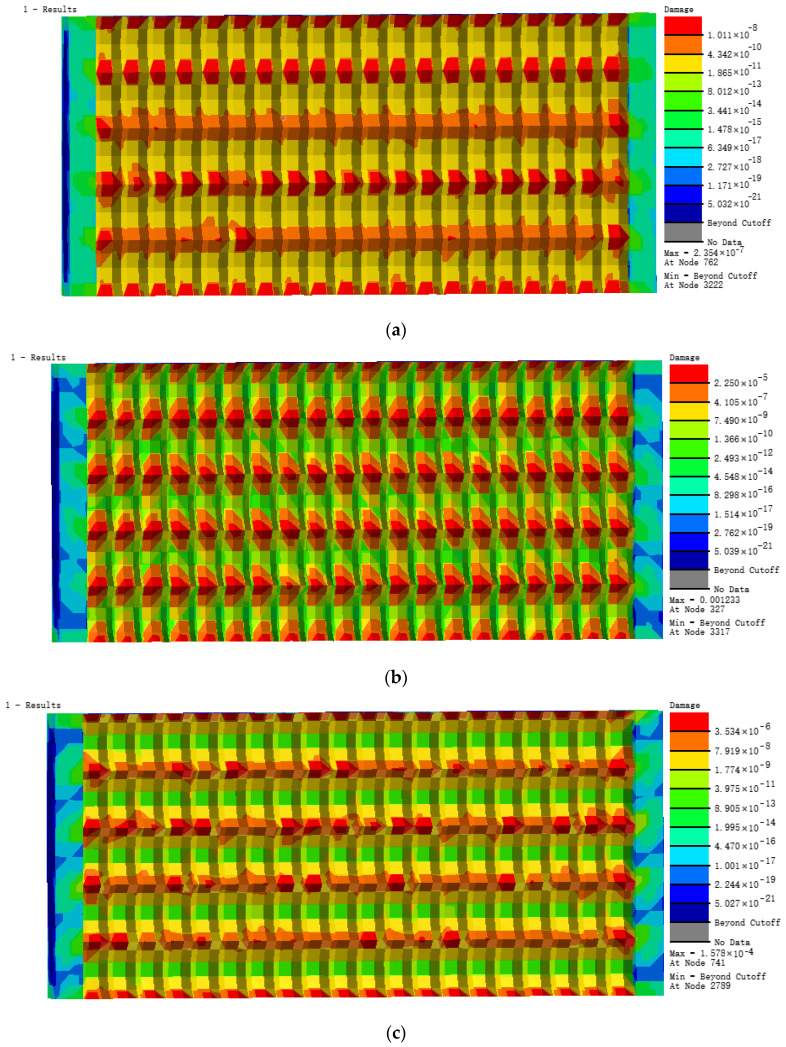

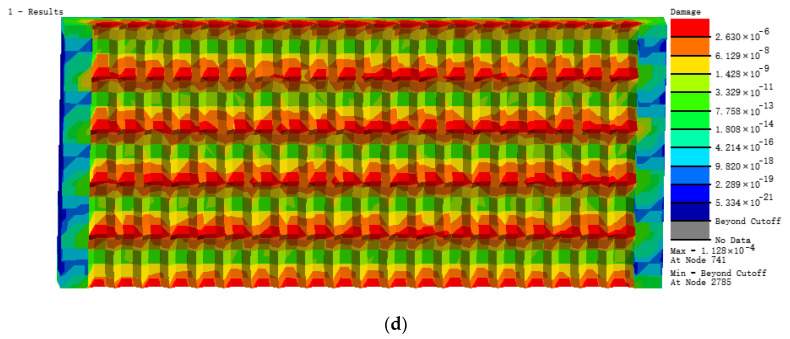
Nephograms of fatigue damage. (**a**) S1 model; (**b**) S2 model; (**c**) S3 model; (**d**) S4 model.

From the analysis of the nephograms of fatigue damage of the four models (Figure 15), we found that their fatigue damage distributions of were considerably different from that of model F2 with a milling depth of 0.3 mm. The fatigue damage distribution was even on the whole model surface without obvious concentration at a milling depth of 0.3 mm, but the damage was obvious concentration over the growing of milling depth. The maximum damage positions are mainly at quadrilateral surface cusps and they are more and more concentrated over the growing of milling depth.

**Figure 16:**
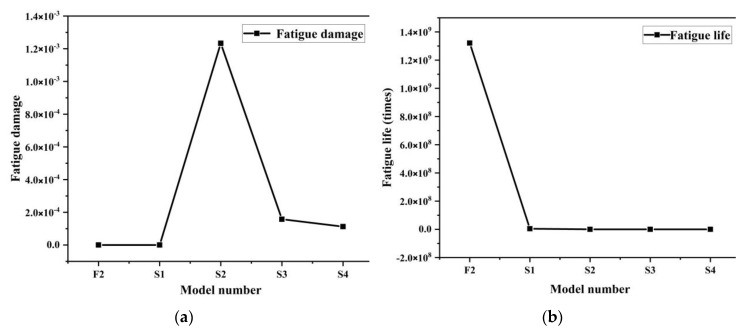
(**a**) Fatigue damage and (**b**) fatigue life of five models.

From the curve of fatigue life of the five models in Figure 16b, we found that fatigue life decreased with increasing milling depth. The stress concentration at surface cusps became increasingly serious under the alternating load, causing fatigue breaking at several surface cusps on the model, which decreased the fatigue life value of the entire model. The fatigue life value of the model sustained 1000 stressing cycles when milling depth increased to 0.5 mm. The specific cloud diagram of fatigue life distribution can be seen in Figure S3. Thus, the milling depth of the biomimetic micro-form of the model should be set to a minimum value to increase the structural fatigue life.

## 4. Fatigue Crack Growth Analysis of Different Bionic Morphology Models

### 4.1. Comparative Analysis of Fatigue Crack Growth Grocess of Different Bionic Morphology Models

#### 4.1.1. Analysis of Fatigue Crack Growth Process in a Hexagonal Bionic Morphology Model

**Figure 17:**
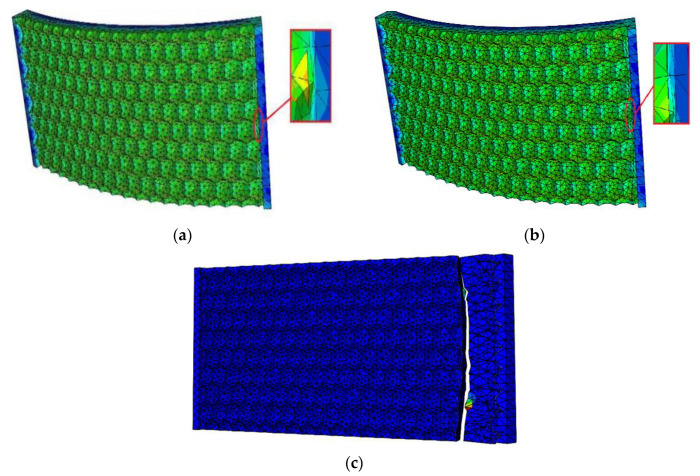
Graph of crack extension of hexagonal model: (**a**) Crack occurrence stage; (**b**) crack extension stage; (**c**) final breaking stage.

Figure 17 shows the fatigue crack growth process in a hexagonal bionic morphology. The crack occurrence location of the hexagonal model is at a surface cusp to the right in the center of the model, and the crack extends to the center and sides from this point, forming a belt of macroscopic crack that cross the intersecting surface. The crack on the surface then extends to the interior of the model gradually until it causes a breakage of the entire model, forming an arc at the fracture, the vertex of which is the point of the initial crack. The reason for the circular arc-shaped integral fracture crack on the hexagonal model is because the hexagonal pits do not block the crack extension direction but enhance the accumulation of cracks from other directions to the surface cusps, which results in circular arc-shaped crack with an irregular serrated form.

#### 4.1.2. Analysis of Fatigue Crack Growth Process in a Quadrilateral Bionic Morphology Model

**Figure 18:**
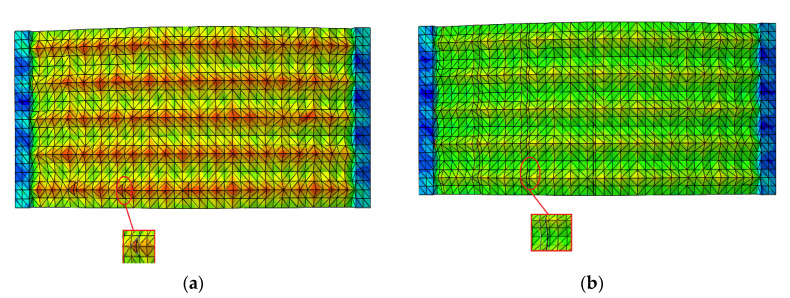

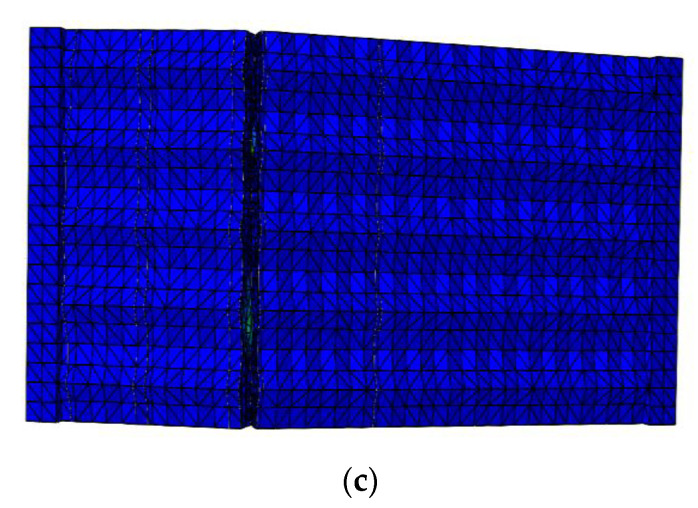
Graph of crack extension of a quadrilateral model: (**a**) crack occurrence stage; (**b**) crack extension stage; (**c**) final breaking stage.

The fatigue breaking of the quadrilateral model, is shown in Figure 18 and, differs from the hexagonal model. Cracks appear at different surface cusps at the same time during the crack occurrence stage; then, cracks extend upwards from the cracking points, forming a straight line of regular macroscopic cracks that cross the whole intersecting surface. Cracks do not extend downward at the same time during the crack extension stage; however, they form regular fracture edges at the crack location in the middle of the model. The formation of regular straight line shaped cracks in the quadrilateral model is due to the obvious effect of quadrilateral pits restraining cracking and controlling the direction of cracks, meaning that it blocks the accumulation of cracks from other directions to the cusps and creates regular fractures that form at the crack location. This greatly improves the fatigue crack resistance of the model material. Previous experiments verified that rectangular pits have a crack restraint effect [33].

### 4.2. Comparative Analysis of Fatigue Crack Growth Characteristic Parameters of Different Bionic Morphology Models

**Figure 19:**
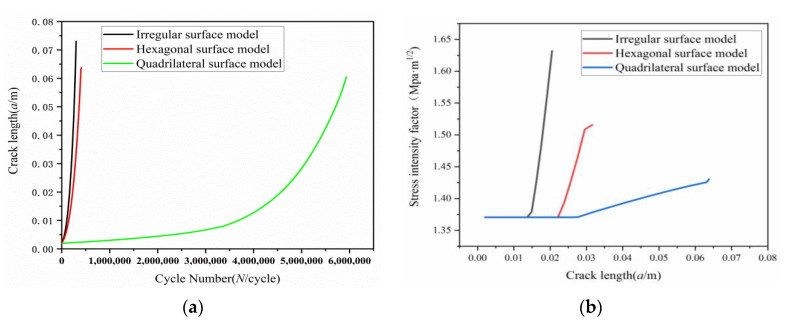
Comparison graph of fatigue feature parameter of three biomimetic form models: (**a**) An a-N curve of fatigue crack extension of three biomimetic form models; (**b**) relation curve of stress intensity factor and crack length.

From the analysis of an a-N curve of fatigue crack extension of three biomimetic form models shown in Figure 19a, it can be seen that the velocity curve of fatigue crack extension of three biomimetic forms is basically in accordance with pairs fatigue extension velocity, which shows a linear increasing trend. The crack extension velocity of irregular models is fastest over the growth of stress cycling times, breaking after 2 × 10^5^ cycles. The crack extension velocity of the hexagonal model is intermediate, breaking after 4 × 10^5^ cycles. The crack extension velocity of the quadrilateral model is the slowest, which can bear 5 × 10^5^ cycles. In the analysis on the relation curve of stress intensity factor and crack length shown in Figure 19b, we found out that the change in stress intensity factor of the irregular model was the fastest over the growth of the crack length, and the stress field at the tip of the crack caused cracks to rapidly extend with instability. The variation curve of the quadrilateral model is most stable, and the stress field at the crack tip increases slowly in the process of crack extension until it goes up rapidly to the critical value in the last phase of crack extension, finally breaking with instability.

In conclusion, the quadrilateral biomimetic form model has the longest fatigue life, lowest crack extension velocity rate and lowest variation velocity rate of stress intensity factor under the same analysis conditions, which is consistent with previous results.

## 5. Conclusions

Based on numerical simulation analysis, we studied the influence of the biomimetic pit form of hardened steel model surfaces on fatigue features and proposed the optimal biomimetic function surface with best fatigue resistance. The following conclusions were obtained:

(1) The optimal form with the highest fatigue resistance is the quadrilateral pit form of the biomimetic dung beetle body based on our comparison of feature parameters such as fatigue life and fatigue damage of three biomimetic forms: the quadrilateral form of a biomimetic dung beetle body, hexagonal form of a biomimetic desert viper abdomen, and an irregular form.
(2) The surface micro dimensions of the quadrilateral form can be changed by alternating milling parameters. We concluded that the model with row spacing of 0.4 mm, feed space of 0.2 mm and milling depth of 0.3 mm provided the optimal fatigue resistance. The dimensions of the quadrilateral formed by the milling process is highly similar to that of a dung beetle body with length-width ratio of 2:1.
(3) The fatigue resistance of the model decreases dramatically with increasing milling depth. The milling depth should be reduced to increase structural fatigue resistance after the process parameters, like row spacing and feed rate, are fixed.
(4) The fatigue resistance of different bionic morphology models was analyzed from the perspective of fatigue crack growth, and typical crack growth modes were obtained for different bionic morphologies, among which the quadrilateral bionic morphology exhibited the best fatigue crack growth inhibition. From the point of view of the characteristic parameters of fatigue crack propagation, we conclude that the quadrilateral pits on the surface of a dung beetle have better fatigue resistance.

## Figures and Tables

**Table 1:** Chemical composition of Cr12MoV hardened steel [31].

Element	C	Cr	Mo	V	Mn
Content	1.45–1.47	11–12.5	0.4–0.6	≤0.3	≤0.4
**Element**	**Si**	**Ni**	**Cu**	**S**	**P**
Content	≤ 0.4	≤0.25	≤0.3	≤0.03	≤0.03

**Table 2:** Physical characteristics of Cr12MoV hardened steel [32].

Elastic Modulus E (GPa)	Poisson’s Ratio ν	Density (kg/m^3^)	Yield Strength (MPa)	Tensile Strength (MPa)
180	0.30	7750	610	850

**Table 3:** Milling parameters of quadrilateral structures with row spacing changes.

No.	Model Code	Feed Space *f_z_* (mm)	Row Spacing *a_e_* (mm)	Milling Depth *a_p_* (mm)
1	A1	0.4	0.1	0.3
2	A2		0.2	
3	A3		0.3	
4	A4		0.4	
5	A5		0.5	
6	A6		0.6	

**Table 4:** Milling parameters of quadrilateral structures over feed space changes.

No.	Model Code	Row Spacing *a_e_* (mm)	Feed Space *f_z_* (mm)	Milling Depth *a_p_* (mm)
1	F1	0.4	0.1	0.3
2	F2		0.2	
3	F3		0.3	
4	F4		0.4	
5	F5		0.5	
6	F6		0.6	

**Table 5:** Milling parameters of quadrilateral models with different milling depth.

No.	Model Code	Feed Space *f_z_* (mm)	Row Spacing *a_e_* (mm)	Milling Depth *a_p_* (mm)
1	S1	0.2	0.4	0.35
2	S2			0.40
3	S3			0.45
4	S4			0.50

## Supplementary Materials

The following are available online at https://www.mdpi.com/1996-1944/13/18/4130/s1: Figure S1: nephograms of fatigue life. (a) A1 model; (b) A2 model; (c) A3 model; (d) A4 model; (e) A6 model, (f) A5 model, Figure S2: nephograms of fatigue life. (a) F1 model; (b) F2 model; (c) F3 model; (d) F4 model; (e) F6 model, (f) F5 model, Figure S3: nephograms of fatigue life. (a) S1 model; (b) S2 model; (c) S3 model; (d) S4 model.
