# Supplementary material for: Study on Fatigue Characteristics of Bionic Functional Surface of Hardened Steel
Source: Materials (Basel). 2020 Sep 17;13(18):4130. doi: 10.3390/ma13184130 (PMC7560293; doi:10.3390/ma13184130)
Supplement: Supplementary file 1 [file materials-13-04130-s001.pdf]

materials

Supplementary materials

Fatigue Characteristics of Bionic Functional Surface of Hardened Steel

Youzheng Cui [1,2], Minli Zheng [1], Wei Zhang [1,*], Ben Wang [1], Yonglei Sun [1] and Weiran Wang [1]

1 Key Laboratory of Advanced Manufacturing and Intelligent Technology, Ministry of Education , Harbin University of Science and Technology, Harbin 150080, China; cuiyouzhenghust@163.com (Y.C.); minli@hrbust.edu.cn (M.Z.); wangbenhust@163.com (B.W.); sunyonglei18201@163.com (Y.S.); 15245201103@163.com (W.W.)

2 School of Mechanical and Electronic Engineering, Qiqihar University, Qiqihar 161006, China

* Correspondence: weizhanghust@yeah.net; Tel.: +86-130-1900-8449

Received: 24 July 2020; Accepted: 9 September 2020; Published: date

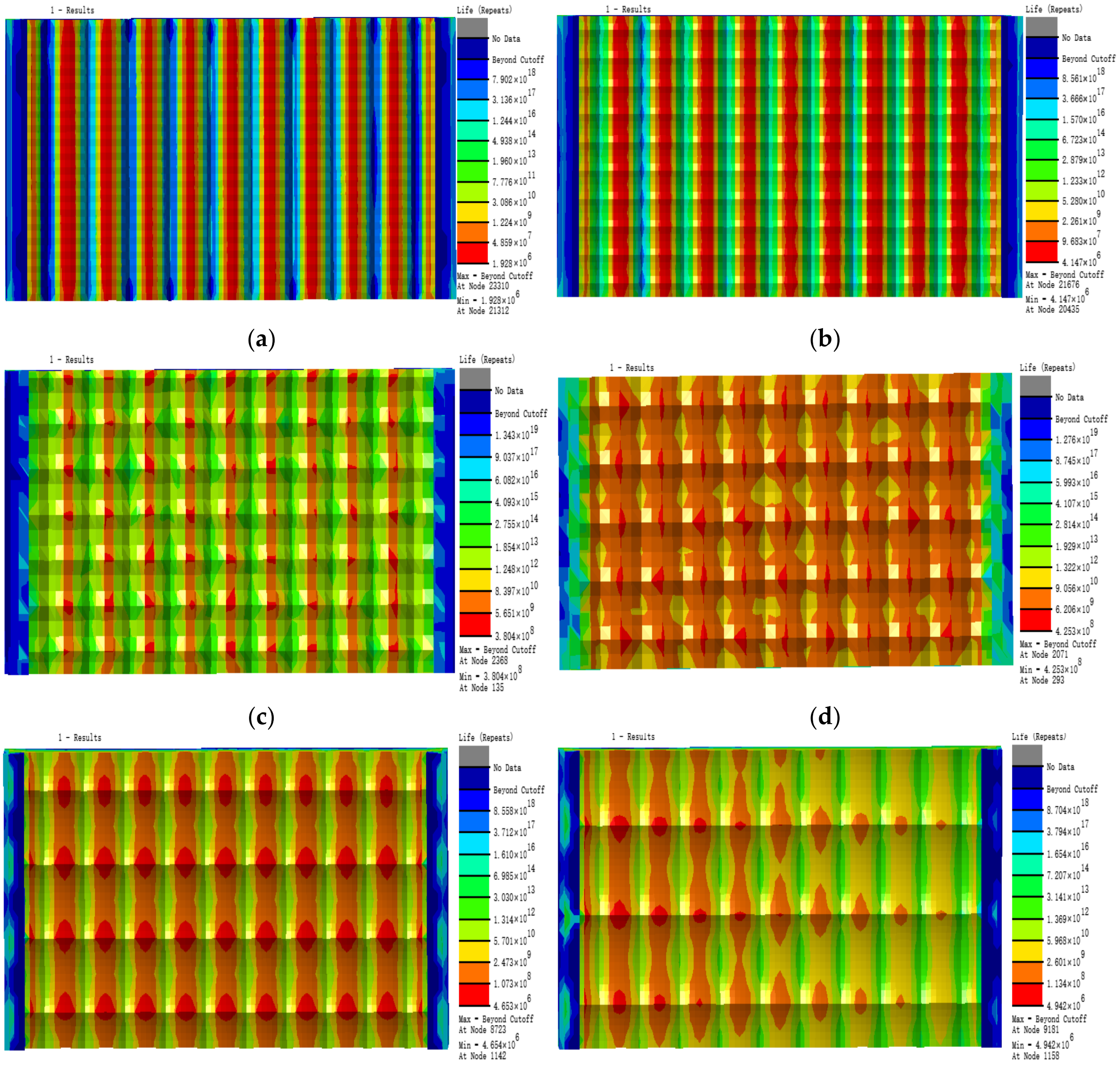

Figure S1. Nephograms of fatigue life. (**a**) A1 model; (**b**) A2 model; (**c**) A3 model; (**d**) A4 model; (**e**) A6 model; (**f**) A5 model.

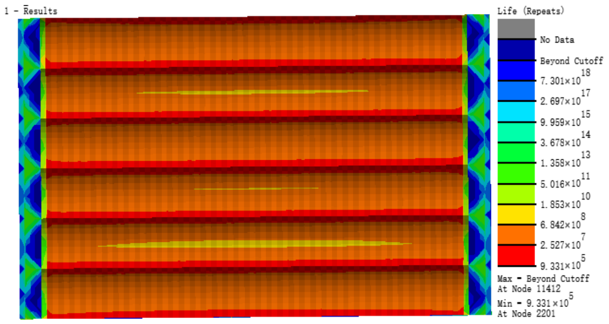

(**a**)

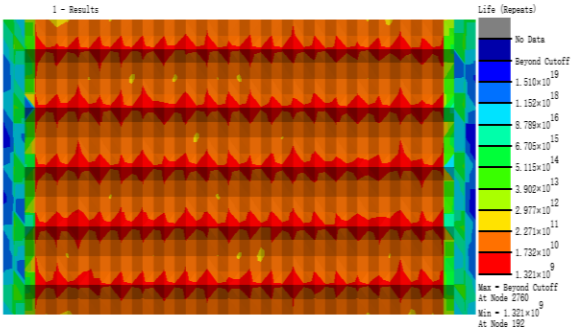

(**b**)

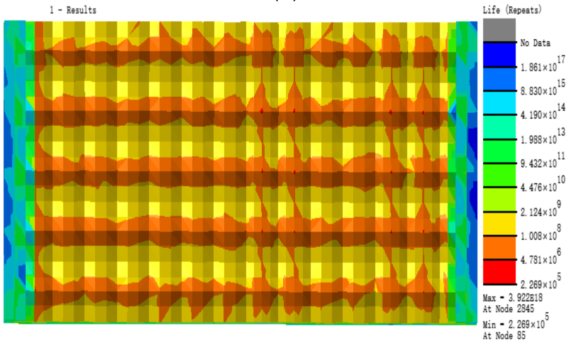

(**c**)

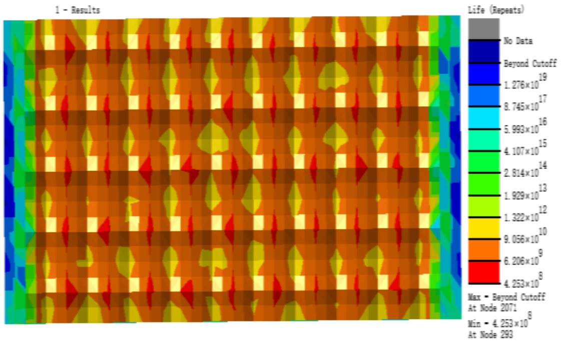

(**d**)

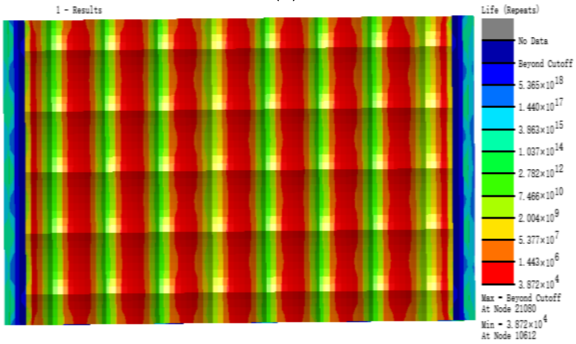

(**e**)

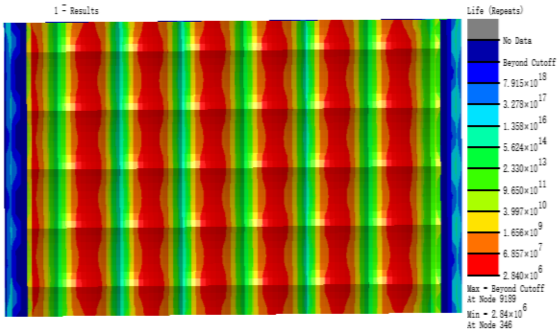

(**f**)

Figure S2. Nephograms of fatigue life. (**a**) F1 model; (**b**) F2 model; (**c**) F3 model; (**d**) F4 model; (**e**) F6 model; (**f**) F5 model.

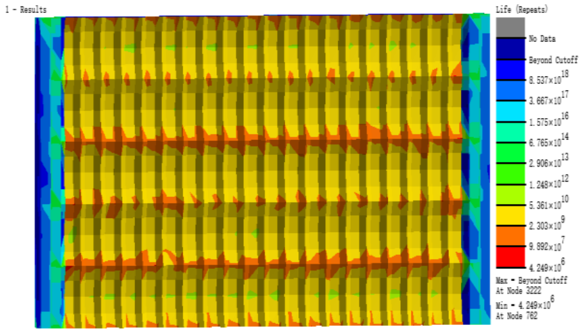

(**a**)

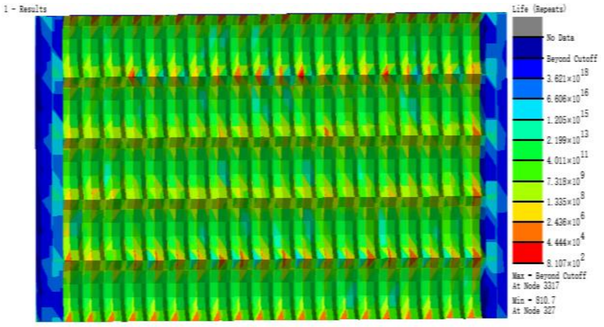

(**b**)

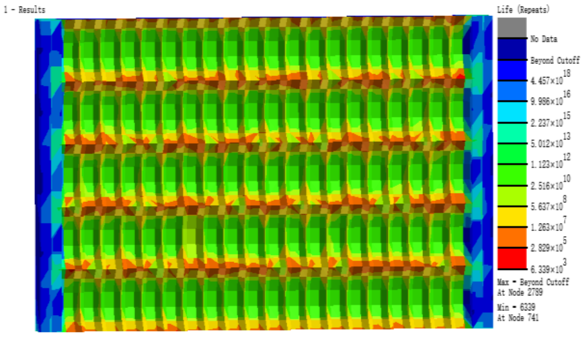

(**c**)

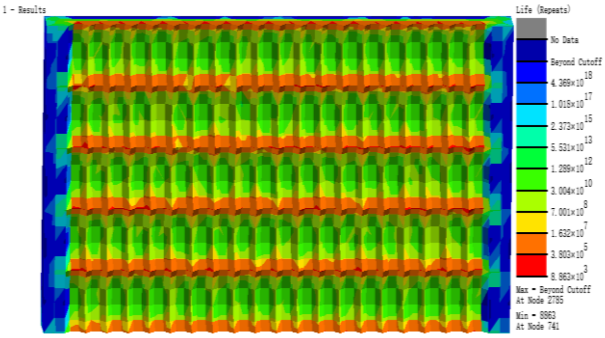

(**d**)

Figure S3. Nephograms of fatigue life. (**a**) S1 model; (**b**) S2 model; (**c**) S3 model; (**d**) S4 model.

© 2020 by the authors. Submitted for possible open access publication under the terms and conditions of the Creative Commons Attribution (CC BY) license (http://creativecommons.org/licenses/by/4.0/).
